# Identifying Medication Review Topics to Be Documented in a Structured Form in Electronic Health Record Systems: Delphi Consensus Survey

**DOI:** 10.2196/70133

**Published:** 2025-05-06

**Authors:** Tanja Lindholm, Noora Lias, Kirsi Kvarnström, Anna-Riia Holmström, Terhi Toivo, Marjo Uusitalo, Harri Nurmi, Marja Airaksinen

**Affiliations:** 1 Clinical Pharmacy Group, Division of Pharmacology and Pharmacotherapy Faculty of Pharmacy University of Helsinki Helsinki Finland; 2 HUS Pharmacy Helsinki University Hospital Helsinki Finland; 3 HUS Internal Medicine and Rehabilitation Helsinki University Hospital Helsinki Finland; 4 Hospital Pharmacy, Wellbeing Services County of Pirkanmaa Tampere University Hospital Tampere Finland; 5 Innovation and Development Unit Istekki Ltd Kuopio Finland; 6 Faculty of Medicine and Health Technology Tampere University Tampere Finland; 7 Finnish Medicines Agency Fimea Helsinki Finland

**Keywords:** medication review, structured documentation, information management, electronic health record, patient information, medication information, medical informatics

## Abstract

**Background:**

Poor data transfer and interoperability between electronic health record (EHR) systems has been a challenge hindering availability and usability of patient information in clinical practice and evidence-based decision-making. To improve data transfer and interoperability, patient information should be documented in a structured format. This also applies to medication-related patient information and results of the interventions, such as medication reviews (MRs), to individually optimize medication regimens, especially in older adults.

**Objective:**

This study aimed to identify what information obtained from MRs should be documented in a structured form in EHRs at a national and organizational level.

**Methods:**

The study was conducted as a 3-round Delphi consensus survey in 2020. The electronic survey was based on a comprehensive inventory of international and national MR procedures in various settings. Expert panelists (N=41) independently assessed which topics should be documented in a structured form in EHRs. The interprofessional panel (N=41) consisted of 12 physicians, 13 pharmacists, 10 nurses, and 6 information management professionals (participation rate 66%-76% in rounds 1-3; consensus limit set at 80%). The responses were analyzed quantitatively and qualitatively.

**Results:**

Consensus was reached on 97.3% (108/111) of predetermined topics to be documented in a structured form in EHRs. Of these, 39 concerned the MR process, 25 related to potentially drug-induced symptoms, 11 related to burden of risks for adverse drug effects, 12 related to laboratory tests and other test results, 12 related to medication adherence, and 9 related to the use of intoxicants. The patient’s blood pressure (mean 4.85, SD 0.53; on a Likert scale 1-5), kidney function (mean 4.81, SD 0.56), and risk of bleeding (mean 4.81, SD 0.56) were ranked as the 3 most important topics to be documented in a structured form. The panel reached a consensus that the information obtained from MRs should be made available to all health care professionals in the national digital repository for patient data and to patients to some extent.

**Conclusions:**

The interprofessional expert panel strongly agreed on the results of the MRs that should be documented in a structured form in EHRs and made available to both health professionals involved in care teams and patients themselves.

## Introduction

### Background

Health care across the world is undergoing digitalization with the goal of well-integrated systems leading to improved work efficiency and patient safety [1,2]. However, the transition phase toward well-functioning digital systems is characterized by fragmented, immature medical records that have become a major challenge and patient safety risk in various health care functions and settings [3-9]. The key issue is that patient information is collected and documented in numerous local and national data systems that do not communicate or enable data access or data transfer between social and health care organizations or even within an organization. This siloed, fragmented information is difficult to share, blurring the overall picture of the treatment and making it difficult for any care team member to take full responsibility for the patient’s care coordination [4,10]. Thus, standardized terminology and operating models are needed for documenting patient information in a structured format to enable data transfer and interoperability between health care units, regardless of the organization or electronic health record (EHR) system [11-17]. This would also enable the secondary use of patient data when estimating the effectiveness of care or future need for health care services.

The same kind of challenges with fragmented patient information apply to documenting patient information on medication regimens to EHRs which has led to shortcomings and safety risks in managing and obtaining up-to-date medication information [18]. In medication risk management, medication reviews (MRs), that is, a structured evaluation of a patient’s medication regimen with the aim of optimizing medicines use and improving health outcomes, are an important strategy for identifying and solving medication-related risks and problems [19-23]. Traditionally, MRs have been a physician’s duty, but practices where other care team members, including pharmacists, contribute to MRs have become more common globally [24-26]. Collaborative MRs have increased the need for a common electronic platform, where MR information can be documented in such a way that it is up to date and available to all health care professionals involved in the medication use process [1,27,28].

### Objective

Structured documentation offers a possible solution to these issues [12-14,16]. To implement structured documentation of medication information, an understanding of what MR information should be available in a structured form is needed. However, no previous studies were found that defined MR information necessary to be documented in a structured form. Therefore, this study aimed at identifying what patient information obtained from MRs should be documented in a structured form in EHRs at a national and organizational level.

## Methods

### Context

The Finnish health care system serves a population of 5.5 million inhabitants and is mainly based on public health care, complemented by private and occupational health care [29]. The services are divided into primary and specialized health care services. The social and health care system is undergoing a major reform that officially started at the beginning of 2023 after a long preparatory phase [30]. Since then, 21 well-being services counties, the City of Helsinki, and the HUS Group (the joint authority for Helsinki and Uusimaa responsible for organizing specialized health care in the Uusimaa region) have been responsible for organizing public social and health care services. The key objective of the reform is improving the availability and accessibility of social and health services, for example, through digitalization of care pathways and patient information. The reform also concerns structures and processes needed for ensuring rational pharmacotherapy as part of patient care [29,31]. Availability of up-to-date electronic medication information and information transfer have been prioritized as the most important issues to be developed in this respect [29-32].

Use of information technology in prescribing medicines, processing prescriptions, and making medicines information available has been a routine practice in Finland since the 1980s when the first technology-based solutions were launched and nationally implemented [33-35]. The deployment of the national digital repository for electronic patient data Kanta since 2010 is one of the most prominent milestones toward electronic patient and medication information management in a closed-loop manner [29,33,36,37]. It enables electronic prescribing and centralized archiving of electronic patient data and data transfer between different health care providers regardless of local EHR. Electronic prescribing became mandatory by law in 2017 [38]. The Finnish Institute for Welfare and Health (THL) is nationally responsible for the definition, distribution, and harmonization of the structures of patient information [39,40]. Thus, it has played a crucial role in the development and harmonization of medication information management in EHRs by describing the target state of medication information documentation which has emphasized the importance of structured documentation. Structured documentation is also increasingly adopted in Kanta and EHRs to address shortcomings regarding data transfer and interoperability as patient and medication information is still stored in hundreds of separate systems [39-41].

Among the driving forces for the ongoing health and social services reform in Finland are the challenges in meeting the service needs of one of the fastest aging populations in the world [29,42]. Aging has increased multimorbidity and complexity of medication treatments, also increasing medication-related risks and problems [4,20,43-48]. Therefore, collaborative MR practices have been developed in Finland since 2005 as a measure to optimize medication treatments individually and to avoid preventable risks and harms [20,23,48-50]. As a result, the medication management process has become increasingly interprofessional, extending the need to share medication-related information from electronic prescriptions to follow-up information on the implementation of the medication regimen.

### The Delphi Consensus Method

#### Overview

This study applied the Delphi consensus method, which is a qualitative survey-based method gathering views of experts with the aim of reaching consensus on the studied topic [51-54]. The expertise and anonymity of the expert panelists, the iteration of research arguments, and the feedback given to the experts on the results of the previous round are the method’s key characteristics. The method has been widely used in health services research for developing treatment recommendations, quality standards and indicators, risk-management tools, and integrating health service systems [21,55-65]. For this study, 3 Delphi rounds were carried out as electronic surveys in September to December 2020 using the eDelphi software developed by Metodix Ltd [66]. The process was led by an 8-member research group consisting of experienced pharmacy professionals familiar with health care information systems, EHR development, IT architecture, and collaborative MRs in various operating environments, having also expertise in Delphi research methodology. The study is reported in alignment with the guidance on Conducting and REporting DElphi Studies (CREDES; Multimedia Appendix 1).

#### Expert Panel

The interprofessional expert panel consisted of the following 41 members recruited by the research group from different parts of Finland: 12 physicians, 13 pharmacists, 10 nurses, and 6 information management professionals. The recruitment had characteristics of snowball sampling, as the selected experts were invited to recommend other professionals as potential members of the expert panel. The primary criterion for the experts was MR expertise. The recruited experts needed to have experience of using patient and medication information in practice, enter MR information into data systems, and understand the possibilities and limitations of structured documentation in EHRs.

Another primary target group of the study were information management professionals (n=6) from different national authorities who had experience in developing EHR systems or other national health information systems. Many of them also had been working as health care professionals, and therefore, understood the importance of structured documentation in EHR systems, bringing invaluable insights to this study. The physicians (n=12) came from various health care organizations and specialties (eg, geriatrics, acute medicine, and oncology), national authorities, and organizations developing EHR systems. The pharmacists (n=13) came from various health care organizations, national authorities, universities, community and hospital pharmacies, and organizations specialized in developing EHR systems. Similarly, the nurses (n=10) were from different areas of health care, working in emergency and geriatric care, some having special expertise in patient safety as well as developing EHRs and closed-loop medication processes.

EHR vendors were excluded from this study as the researchers did not want commercial interests or conflict of interests influencing the results. Furthermore, this study was primarily aimed at representing the mindset of public-sector health care professionals who conduct MRs because public health care is the mainstay in Finland.

#### Identifying MR Topics for the First Delphi Round

**Figure 1:**
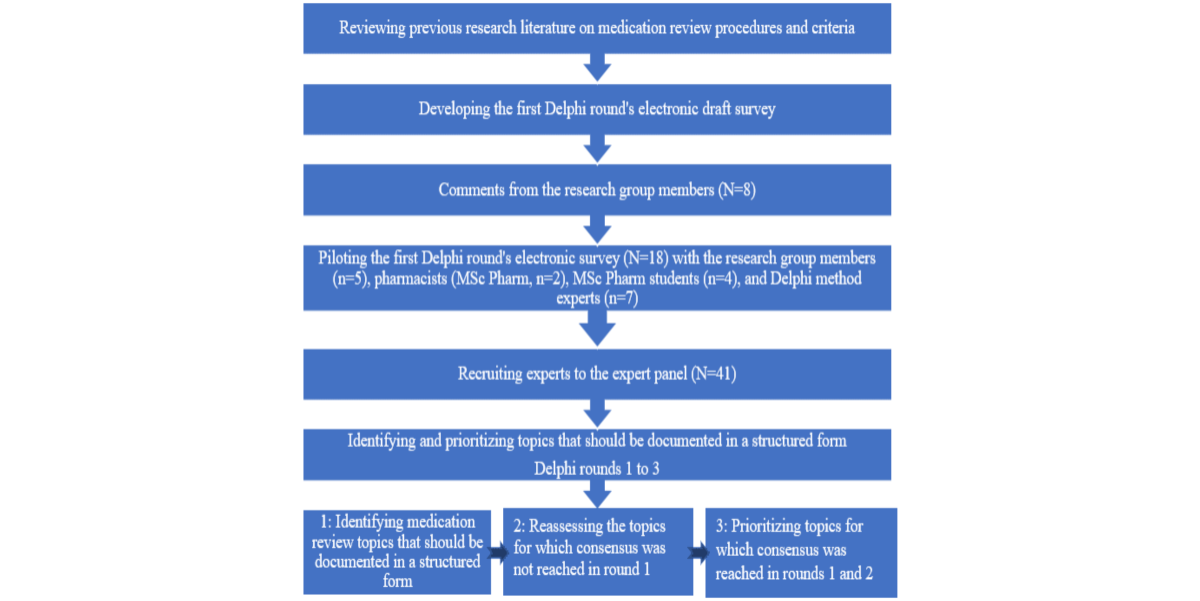
The study outline aimed at identifying medication review topics to be documented in a structured form in electronic health records.

MR topics for the first electronic Delphi round survey were identified by comprehensively reviewing literature on national and international MR definitions, procedures, practices, and inventories in various settings [20,22,24-26,48,50,67-80] (Figure 1). In addition, internationally recognized and most widely used criteria of potentially inappropriate medications for older adults and drug-related problems (DRPs) were used [55,56,81-86]. A validated tool for assessing risks for DRPs to be used by practical nurses caring for home-dwelling older adults, and a validated checklist for medication-related risk factors (Lääkehoidon onnistumisen tarkistuslista LOTTA checklist) to be self-assessed by older adults were also used [21,49,87]. The identified MR topics were inductively divided into six categories: (1) MR process topics, (2) potentially drug-induced symptoms, (3) the burden of risks for adverse drug effects, (4) laboratory tests and other test results, (5) medication adherence, and (6) the use of intoxicants.

The first Delphi round’s survey instrument was piloted by 18 experts other than panel members before the actual round to ensure the face and content validity and usability of the survey [88]. The results of the pilot were analyzed and compared by the 2 main researchers (TL and NL) who then presented their findings to the research group. On the basis of the pilot, a few minor changes were made to the survey. Examples of liver function tests (ALAT, ASAT, and GT), examples of electrolytes (potassium and sodium), and the orthostatic test were added to laboratory tests and other test results. In addition, the wording of 1 item was modified (“The medicine user is afraid of possible adverse effects” was modified into “The medicine user expresses concern about possible adverse effects”).

The panelists could choose from the following structured answer options when assessing each of the presented topics during rounds 1 and 2: (1) “Yes, as presented”; (2) “Yes, but modified”; (3) “Not at all”; and (4) “Cannot say.” In each round, the panelists were asked to justify their answer in an open comment field if they chose to answer with option 3, “Not at all.” The panelists were asked to comment and suggest changes if the presented topic was suitable but after modifications. The panelists were encouraged to provide comments and justifications for their choices in the open comment fields and to suggest new additional potentially missing topics.

#### Delphi Round 1

**Figure 2:**
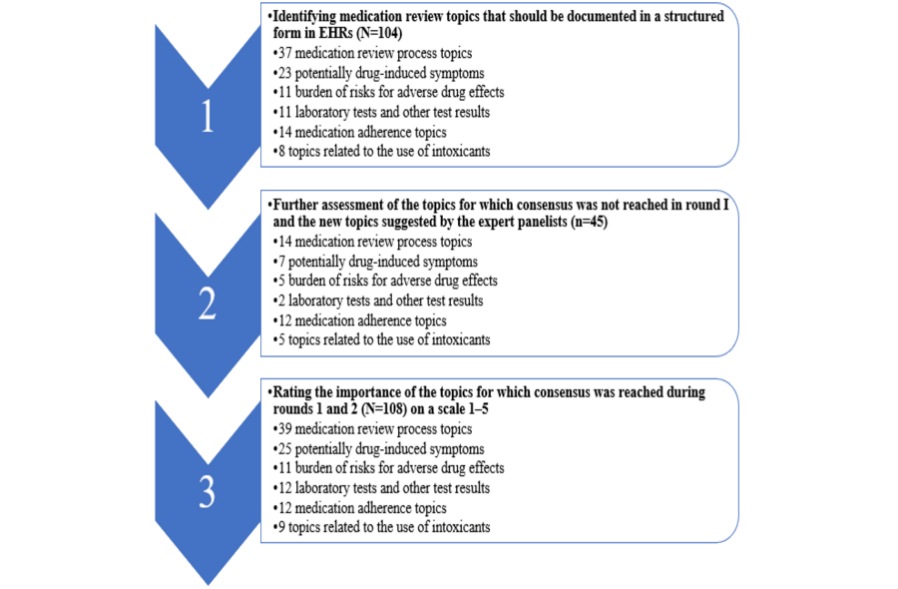
The distribution of medication review topic input by category for Delphi rounds 1 to 3. Rating the importance of the topics in the third Delphi round was conducted on a Likert scale 1 to 5 (5: important topic, 4: moderately important topic, 3: cannot say, 2: moderately unimportant, and 1: unimportant topic). EHR: electronic health record.

During the first Delphi round, the panelists were asked to assess which MR-related patient information and other patient data (N=104) should be documented in a structured form in EHRs (Figure 2).

#### Delphi Round 2

The second Delphi round consisted of the topics for which consensus was not reached during the first round. New topics suggested by the panelists during the first round were also added. Thus, a total of 45-MR topics were presented to the expert panelists during the second round (Figure 2). In addition, the expert panelists were asked to assess if MR-related information should be available in the national Kanta and MyKanta services. MyKanta is a web-based part of Kanta through which citizens can access their health information, patient data, prescriptions, and laboratory test results documented by health care providers [89,90].

#### Delphi Round 3

During the third round, the expert panelists were asked to rate the importance of each of the topics for which consensus was reached during the first and second rounds (N=108) on a Likert scale from 1 to 5 (Figure 2). The importance of prioritization was emphasized, as not all topics can be structured at the same time. In addition, the expert panelists were asked to take the context into consideration, as not every topic is appropriate for every patient.

#### Data Analysis

The responses from each Delphi round were analyzed with Microsoft Excel by the 2 main researchers (TL and NL) who then compared their findings. The results were presented to and discussed with the whole research group that also resolved possible disagreements and approved the survey instrument to be sent for the next Delphi round. The results were reported using descriptive analysis with the main parameters being frequencies and percentages with SDs. Responses to the open-ended questions were analyzed qualitatively using inductive content analysis to modify the topics for the next round [91]. The consensus limit was set at 80% [54,92].

### Ethical Considerations

The study was conducted in accordance with the national guidelines for responsible conduct of research and procedures for handling allegations of misconduct in Finland by the Finnish Advisory Board on Research Integrity [93]. The ethical principles of research with human participants and ethical review in the human sciences in Finland by the Finnish National Board on Research Integrity were followed [94]. According to the guidelines, a review by the research ethics committee and their approval were not required for this study. Participating in the expert panel was voluntary, and written informed consent was received from all expert panelists. The expert panelists were aware that their responses would be treated anonymously and used for research purposes only. The research material is stored in accordance with current regulations, considering the General Data Protection Regulation of the European Union [95].

## Results

### Participation Rates

The results of the study consist of the MR topics for which consensus was reached during the 3 Delphi rounds. The response rates for all respondents (N=41) were 66% to 76% during all 3 Delphi rounds (Table 1).

**Table 1:** The number of experts participating in the 3 Delphi rounds, the number of respondents, and response rates in each round. Response rates varied depending on the question as answering all questions was not mandatory.

Expert panel		Round 1	Round 2	Round 3
**Experts participating in the study, n**				
	All professional groups	41	41	41
	Physicians	12	12	12
	Pharmacists	13	13	13
	Nurses	10	10	10
	Information management professionals	6	6	6
**Experts who answered in each round, n (%)**				
	All professional groups	30-31 (73-76)	27-29 (66-71)	27-28 (66-68)
	Physicians	8 (67)	8 (67)	6 (50)
	Pharmacists	13 (100)	12 (92)	12 (92)
	Nurses	5-6 (50-60)	5 (50)	6 (60)
	Information management professionals	4 (67)	2-4 (33-67)	3-4 (50-67)

### Consensus

**Figure 3:**
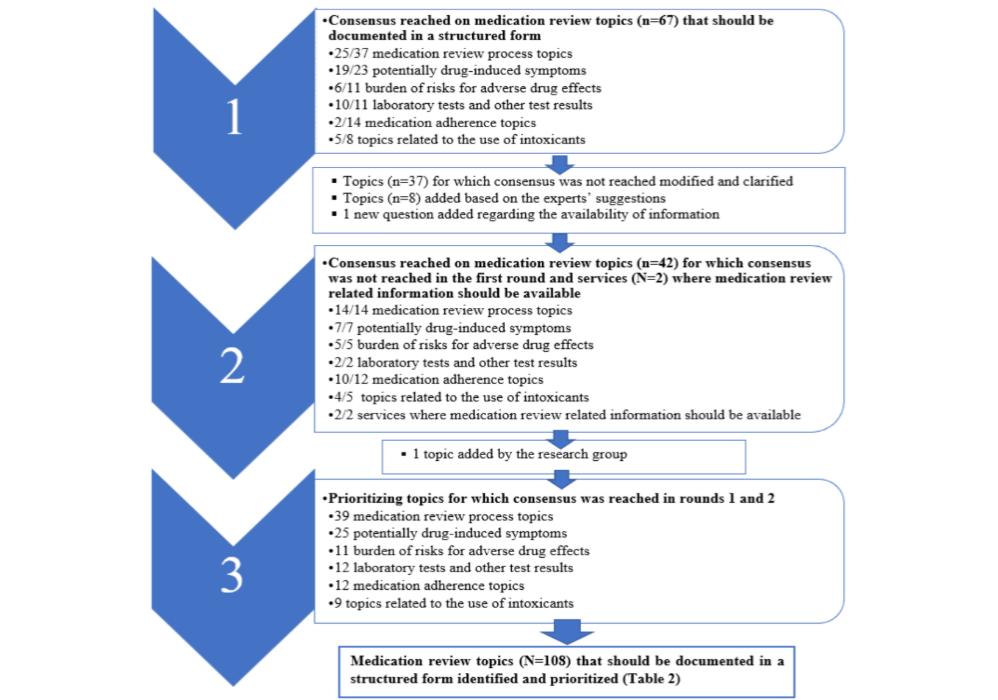
A summary of the results of the Delphi rounds 1 to 3. One of the medication review process topics (duration of drug treatment) was not prioritized in the third round due to technical issues.

Of the 111 topics, the expert panelists reached consensus on 108 (97.3%) topics during the 3 Delphi rounds as shown in Figure 3 and Table S1 in Multimedia Appendix 2. During the first Delphi round, consensus was reached for 67 of 104 (64%) MR topics that the expert panelists assessed should be documented in a structured form in EHRs. Topics (n=37) for which consensus was not reached during the first Delphi round were modified and, if necessary, clarified based on the expert panelists’ comments. For consensus to be reached during the first Delphi round, it was determined based on the number of respondents (n=30-31) that 24 (77%) of the experts had to accept the presented MR topic. The expert panelists reached a consensus on 42 out of 45 (93%)-MR topics during the second Delphi round (Figure 3; Table S1 in Multimedia Appendix 2). Depending on the varying response rate (n=27-29) for each question during the second Delphi round, it was determined that 21 to 22 (77%) of the experts had to accept the presented topic for consensus to be reached. A clear consensus was also reached regarding that the MR-related information should be available in the national Kanta (29/29, 100%) and MyKanta services (25/29, 86%).

### Importance

During the final third round, the expert panelists rated the structured documentation of most of the topics for which consensus had been reached during the previous rounds as important or somewhat important with 92 of the 108 topics (85%) reaching a mean of at least 4.0 (Table S1 in Multimedia Appendix 2; Table 2). The 10 most important topics according to the experts are presented in Table 2.

**Table 2:** The 10 most important medication review items that should be documented in a structured form in the electronic health records. The items are presented in a descending order of the mean ratings and SD for each item as established in the third Delphi round on a Likert scale 1 to 5 (5: important topic, 4: moderately important topic, 3: cannot say, 2: moderately unimportant topic and 1: unimportant topic).

Medication review item	Ratings, mean (SD)
Blood pressure	4.85 (0.53)
Kidney function tests	4.81 (0.56)
Risk of bleeding	4.81 (0.56)
Date of medication reconciliation	4.78 (0.51)
Drug-drug interactions	4.78 (0.51)
Inexplicable bruises, nose bleeds, or dark stools	4.78 (0.58)
Blood glucose level	4.78 (0.58)
International normalized ratio	4.78 (0.58)
Sedative burden	4.78 (0.58)
Renal toxicity burden	4.78 (0.58)

## Discussion

### Principal Findings

A large and committed interprofessional expert panel was obtained for this Delphi study, which enabled the identification and prioritization of MR topics that should be documented in a structured form in EHRs. To our knowledge, this is the first reported study aimed at systematically identifying these topics at the national level. Interprofessional studies involving professionals participating in patient care are essential when developing EHR systems and defining their contents. This approach ensures the views of different professional groups are integrated, enhancing the usability and compatibility of systems. The Delphi method proved to be suitable for this purpose. The structured documentation of MR topics in EHRs was perceived as important or somewhat important in most of the presented topics, including basic information about the patient’s health status, medication regimen, outcomes of the MR process, potentially drug-induced symptoms, burden of risks for adverse drug effects, results of laboratory tests and other tests, adherence, and the use of intoxicants, such as alcohol.

On the basis of the results, the patient’s blood pressure, kidney function, and risk of bleeding were clearly considered the 3 most important topics to be documented in a structured form (Table 2). Supported by previous research, the experts raised their concern that especially the estimated glomerular filtration rate (eGFR) value is often ignored even though it essentially affects the drug and dosage choices especially in older patients [96-99]. The structured documentation of the kidney function could also enable the use of electronic decision support systems in medication management in older adults [100,101]. Our results are in line with the increasing body of evidence indicating the importance of taking the kidney function into account in medicines optimization [102]. However, the eGFR value is not yet routinely and easily available to all care team members involved in the implementation of the drug therapy [27,103,104].

Until now, a major portion of EHR data has consisted of unstructured elements [105,106]. Structured data has typically included patient demographics, vital signs, laboratory results, diagnostic codes, medication prescriptions, dosages, and administration methods, whereas elements such as clinical notes and discharge summaries have been unstructured. However, our results indicate a shift toward EHR systems that facilitate individual optimization of drug treatments. Updating standard terminology within coding systems, such as LOINC (Regenstrief Institute) and RxNORM (National Library of Medicine), so that they would enable embedding the identified MR items with Health Level 7 Fast Healthcare Interoperability Resources solutions, would thereby be essential [107-109]. Even though many of the clinical observations related to MRs and basic information about the patient and medicines in use are expressed and documented in standard terminology, there is still a lack of specific terminology for medication use aspects. Such aspects include confirming the patient specific therapeutic suitability and the appropriateness of medications, dosage regimen, administration route, and discontinuation of the medication (deprescribing). Once embedded within Fast Healthcare Interoperability Resources profiles, the exchange and use of more patient-specific MR data would be enabled and made electronically available to care team members, ideally also to community pharmacists while dispensing, counseling, and reviewing medications or contributing in any way to the appropriate and safe medication use [110-112]. This is particularly important considering aging populations leading to increased comorbidity, polypharmacy, and complex treatments that need to be managed in clinical practice [20,43-48,113-115].

Medication adherence topics were rated slightly less important to be documented compared with other datasets. This may be explained by the fact that medication adherence can be considered difficult to measure as it is usually based on patient reporting or claims-based evaluations as proportion of days covered in comparison with more easily measured markers of clinical effect, such as blood pressure or blood coagulation risk [116-119]. On the other hand, adherence as a behavioral issue may be considered complicated to measure due to a lack of valid and reliable measures feasible to be used in routine clinical practice or to be integrated in EHRs or electronic care pathways [120-124]. The adherence items identified in this study address behavioral factors related to knowledge and skills in taking medication, beliefs about consequences in taking medication, and negative emotion and beliefs about capabilities in taking medication [125]. These items could serve as a short set of questions integrated into the EHR to help assess patients’ medication taking behaviors and screen for adherence status. This kind of integrated measure could help to identify contributing factors or even root causes to nonadherence and to help plan effective customized physician communication, which is one of the main factors influencing medication adherence [126]. Medication adherence is a significant factor affecting real-life effectiveness of drug therapies, as only about 50% of patients treat their long-term condition according to instructions [127,128]. It would be useful to integrate medication adherence items with other treatment-related adherence measures, such as diet, physical activity, or other lifestyle changes. This extension may provide a more holistic understanding of adherence and disease management problems, enabling also differentiation of medication adherence issues from other treatment adherence issues. In this respect, EHRs should be developed to enable the documentation and monitoring of adherence status and self-management of the treatment to identify problems that should be solved together with the patient [126,129,130].

The expert panel strongly agreed on having structured MR information in the national Kanta patient information repository, while they commented on several aspects to be considered if the information was made accessible to patients in the MyKanta service. One of the concerns that emerged was the risk of the patients starting independently implementing suggested medication changes without consulting a physician. This concern is in line with physicians’ previous concerns that the patients’ access to their own health information, especially if there is not a physician available to interpret the information, may upset patients and cause wrong interpretations [131-135]. Therefore, previous studies have concluded that not all information should be real-time and that health care professionals should have time to interpret the obtained results and prepare the treatment plans [131,136,137]. However, our results indicate that health care professionals have internalized the positive effects achieved by enabling patient access to their own information. The experts commented that the availability of information in MyKanta could increase the transparency of health care functions and increase a sense of trust. They recommended that the patient could see the final result or a summary of the MR in the MyKanta service but not the possible intermediate stages. These aspects need to be considered when further developing the Kanta and MyKanta services.

Structuring the identified MR topics could further promote the uniformity and standardization of the MR process and its documentation, which have been called for [15,79,138-140]. Organized and easily accessible documentation helps to create an overall picture of the patient’s situation so that those participating in the care provision can more easily make use of the previously collected essential information and notice changes in the patient’s condition making it easier to plan further treatment and discharge [141]. Enhancing the electronic communication between care providers has also proven to be crucial for the large-scale implementation of collaborative MRs [142,143]. Moreover, structured documentation of patient information could contribute to the secondary use: assessing reliably the effectiveness and cost-effectiveness of MRs, and thereby, possibly supporting their large-scale implementation into the routine clinical practice [142,144].

It is, however, important to recognize that although MR practices can benefit from standardization, it is still crucial to maintain flexibility in individualizing MRs according to the context, medical and patient needs, and available resources [25,144,145]. As medication use seldom is black and white, the need for maintaining flexibility also applies to the documentation of MRs. Thus, a combination of freeform text and structured documentation has been called for [146,147]. To capture clinical and contextual information and interpretations about aspects, such as complexity, severity, or uncertainty, a text box for entering a freeform narrative of the clinical note might be included in an EHR to supplement the structured information [148-150]. This may apply to parts of MRs where a significant degree of variation occurs hindering the use of a predefined template. Such areas could be explaining the reasons behind nonadherence and self-management problems, describing how identified adherence issues have been resolved or describing the patient’s past medical history or the history of the present illness or treatment. To effectively structure the identified MR items, EHR design should focus on key aspects, such as clear labeling of structured fields for ease of use, alignment of data entry processes with clinical workflows, and optimization of user interfaces and interoperability [150-153].

### Strengths and Limitations

Our research addresses a very current issue related to the shortcomings of electronic patient information management, particularly information related to medication treatments. The 2 main researchers (TL and NL) thoroughly reviewed previous literature, MR practices, and criteria when preparing for the first Delphi round survey to identify topics that should be documented in a structured form. This was important, because the comprehensiveness and quality of the first round’s survey is critical for a successful Delphi study [51-54]. The comprehensiveness of the preparatory work is supported by the fact that the expert panel proposed only a few new topics and found the presented topics appropriate. To avoid important items being excluded in the case of nonconsensus or the assumption that consensus would automatically imply the correct answer, the results of the study were continuously compared with scientific evidence and the clinical expertise of the research group that ultimately had the final say.

The expert panel was committed and large in number with a geographical and interprofessional representation. The participation rates remained high throughout the Delphi survey, reflecting commitment and interest in the subject. Of the health care professionals involved, pharmacists were most active, possibly making the results slightly pharmacist oriented. However, due to the strong consensus of the expert panelists, it can be assumed that no great distortion occurred. The lack of patients’ perspective can be considered a limitation of this study. However, this was a conscious choice made by the researchers at this point as patients cannot be expected to be able to assess the clinical relevance and importance of the MR items presented in the survey in its current form. As members of the research group who ultimately possessed the final say had experience in IT architecture and EHR development, we believe that the absence of EHR vendors in the expert panel did not affect the quality of our research. Even though the Delphi method has proven to be suitable for these kinds of medication safety and risk management studies, the typical methodological limitations of the Delphi method remain [51-54]. This means that the results represent the views of the expert panel and are dependent on the selection criteria and study process. As the panel consisted of Finnish experts working in the Finnish context, the results as such cannot be directly generalized beyond Finland, but they can be indicative for other countries and their EHR development.

### Practical Implications

Digital patient information management applications play an increasingly important role in the planning, implementation, monitoring, and optimizing of pharmacotherapies among other health care services. In this study, a carefully built comprehensive list of MR topics was developed based on international literature. This gives EHR developers and clinicians a starting point for further prioritization of topics suitable for different operating settings for both primary and secondary use, even though MR processes in different countries differ in terms of the available patient data, patient involvement, and the purpose of the MR. Such a prioritization has not yet been internationally conducted to any greater extent [139]. Our study can contribute to expanding the scope of the more traditionally reviewed MR topics toward a more comprehensive patient-centered direction as well as standardizing the MR process and its documentation.

Structuring the MR process could also contribute to the effectiveness of MRs and identifying patients benefitting from MRs, thus supporting the large-scale integration of MRs into the standard care [142]. The topics related to drug treatment monitoring could prove significant in this regard, as these often represent components that influence the outcomes of MRs [141]. This combined with the fast advances in integrating artificial intelligence (AI) tools for natural language processing of free text into EHRs would significantly enhance the MR process [154]. This will extend the function of the medication-related documentation in EHRs from a technical data repository toward a prospective clinical decision support system and medication risk management system. The results of this study clarify the issues encountered during the implementation of drug treatments, identify the components of MRs, and highlight which of these components represent critical measures that should be monitored in the patients’ condition during treatment. In a well-organized information system, these identified factors are quite likely to improve therapeutic outcomes, reduce risks and problems, and increase the efficiency of drug treatment implementation processes.

### Further Research

In this study, a strong consensus emerged for the structured documentation of a large number of MR topics. The next step in ensuring the clinical feasibility and practical implementation of structured documentation is to define how the information identified in this study should be structured in practice so that the documentation does not become too laborious and time-consuming. Therefore, automated clinical documentation and AI applications should be developed in such a way that compiling and interpreting the information is automated, reliable, and covers the essential information regarding medication treatments [139,155]. Since not all information can be structured at once, further prioritization is needed. This would enable the use of the structured information for secondary purposes such as identifying patients at risk of developing DRPs and thus benefit from MRs by developing alerts and using AI in automatic screening. Starting with the most prioritized information related to blood pressure, eGFR and the risk of bleeding would seem logical. Involving patients and EHR vendors is an essential prerequisite to ensure that the perspectives and priorities of patients, especially what comes to adherence items, are taken into account in the further development and practical implementation of the structured documentation of MR items. Furthermore, it must be defined which MR information should be made available to patients and what information the patients themselves could document in electronic patient information systems. However, pilots are needed to test new kinds of digital documentation and their use in the medication management system.

### Conclusions

The committed interprofessional expert panel had a strong consensus on a wide range of MR-related information that should be structurally documented in electronic patient information systems. Further prioritization of the information identified in this study is needed. The expert panel also had a consensus that the MR information should be available to all health care professionals involved in a care team and the patients themselves with some reservations.

## Appendix

Multimedia Appendix 1 Guidance on Conducting and Reporting of Delphi Studies.

Multimedia Appendix 2 The identified medication review (MR) topics (N=108) that should be documented in a structured form in the electronic health record and the MR topics (n=3) for which consensus was not reached.

## Abbreviations

AI: artificial intelligence
CREDES: Conducting and REporting DElphi Studies
DRP: drug-related problem
eGFR: estimated glomerular filtration rate
EHR: electronic health record
MR: medication review

## Data Availability

The datasets generated or analyzed during this study are available from the corresponding author on reasonable request.
