# Supplementary material for: Identifying Medication Review Topics to Be Documented in a Structured Form in Electronic Health Record Systems: Delphi Consensus Survey
Source: J Med Internet Res. 2025 May 6;27:e70133. doi: 10.2196/70133 (PMC12093078; doi:10.2196/70133)
Supplement: Multimedia Appendix 2 [file jmir_v27i1e70133_app2.pdf]

This is a Multimedia Appendix to a full manuscript published in J Med Internet Res. For full copyright and citation information see http://dx.doi.org/10.2196/jmir.70133

Table S1. The identified medication review (MR) topics (N=108) that should be documented in a structured form in the electronic health record and the MR topics (n=3) for which consensus was not reached. The topics are presented in a descending order of the mean ratings and SD for each topic as established in the third Delphi round on a Likert scale 1 to 5 (5: important topic, 4: moderately important topic, 3: cannot say, 2: moderately unimportant topic, and 1: unimportant topic), and consensus percentages established in rounds 1 and 2.

Medication review topic that should be documented in a structured form	Round 3, mean (SD)	Round 1, consensus (%)	Round 2, consensus (%)
**Medication review process topics (n=40)**			
Date of medication reconciliation	4.78 (0.51)	30 (97)	—[a]
Drug-drug interactions	4.78 (0.51)	25 (81)	—
Therapeutic duplication	4.74 (0.53)	23 (74)	27 (96)
Appropriateness of medication dosage	4.74 (0.59)	24 (77)	—
Age (years)	4.71 (0.85)	29 (94)	—
Potential adverse drug effects	4.63 (0.56)	25 (81)	—
Making a deprescribing note	4.63 (0.63)	28 (90)	—
Contraindications	4.63 (0.63)	23 (74)	26 (93)
Review of the patient’s complete medication list when prescribing (including medications from all specialties)	4.63 (0.88)	26 (84)	—
Weight	4.61 (0.69)	29 (94)	—
Medicines that require special monitoring (eg, concentration monitoring)	4.59 (0.93)	27 (87)	—
Medicines that have been tried for the patient but found to be unsuitable (+ reason)	4.56 (0.58)	24 (77)	—
Potentially inappropriate medication for older patients	4.56 (0.64)	22 (71)	26 (93)
Planned deprescribing schedule	4.52 (0.75)	27 (87)	—
Drug and excipient allergies	4.52 (0.89)	27 (87)	—
Confirmation of suitability of off-label use[b]	4.44 (0.70)	—	25 (89)
Clinically important drug-food interactions	4.44 (0.75)	17 (55)	24 (86)
Performed medication reconciliation	4.41 (0.97)	24 (77)	—
Suggested medication changes[c]	4.37 (0.79)	—	—
Desired therapeutic effects of medicines	4.33 (0.78)	24 (77)	—
Suitability of medication for children (<12 y)	4.33 (0.83)	20 (65)	26 (93)
Medicines that have been tried to the patient but did not have the desired effect	4.33 (0.83)	25 (81)	—
Suitability of the administration route	4.33 (0.96)	20 (65)	26 (93)
Possible overmedication compared with treatment recommendations	4.26 (1.02)	26 (84)	—
Monitoring schedule[b]	4.26 (1.06)	—	26 (93)
Whether or not monitoring has been carried out	4.19 (1.11)	24 (77)	—
Dosing schedule	4.15 (0.99)	24 (77)	—
Height	4.11 (0.99)	29 (94)	—

Possible undermedication compared with treatment recommendations	4.07 (1.04)	25 (81)	—
Suitability of the dosage form	4.07 (1.11)	21 (68)	28 (100)
Clinically important unwanted effects of drugs on laboratory test results	3.96 (0.94)	18 (58)	25 (89)
Body surface area[d]	3.86 (1.01)	13 (42)	23 (82)
Date of the patient interview or the person responsible for the medication	3.85 (1.20)	29 (94)	—
Pharmacogenomic factors	3.81 (1.04)	17 (55)	24 (86)
Review of the patient's medication list when prescribing (reviewing only medications of one's own specialty)	3.81 (1.33)	20 (65)	25 (89)
BMI (kg/m^2)	3.75 (1.14)	20 (65)	24 (86)
Gender	3.75 (1.38)	29 (94)	—
Health care professional who interviewed the patient or person responsible for the medication	3.30 (1.30)	27 (87)	—
Health care professional who has carried out the monitoring	3.30 (1.32)	24 (77)	—
Duration of the medication treatment[e]	—	24 (77)	—
**Potentially drug-induced symptoms (n=25)**			
Inexplicable bruises or nose bleeds or dark stools	4.78 (0.58)	26 (84)	—
Hypotension (systolic <110 mm Hg)	4.74 (0.59)	26 (84)	—
Repeated falls in the past 12 mo	4.70 (0.61)	26 (84)	—
Urinating difficulties: urinary hesitancy[b,f]	4.63 (0.63)	24 (77)	27 (100)
Whether or not the symptom is new	4.63 (0.79)	27 (87)	—
Mental confusion	4.63 (0.79)	26 (84)	—
Edemas	4.59 (0.64)	24 (77)	—
Extreme fatigue	4.56 (0.64)	25 (81)	—
Rash	4.52 (0.64)	24 (77)	—
Visual impairments	4.52 (0.80)	25 (81)	—
Nausea	4.48 (0.80)	25 (81)	—
Dizziness when standing up (orthostatism)	4.48 (0.94)	25 (81)	—
Memory difficulties	4.44 (0.93)	25 (81)	—
Dizziness	4.41 (0.80)	26 (84)	—
Diarrhea	4.37 (0.79)	25 (81)	—
Prolonged sleeping difficulties	4.37 (0.79)	23 (74)	24 (89)
Walking difficulties	4.37 (1.04)	23 (74)	24 (89)
Muscle pains	4.30 (0.87)	23 (74)	24 (89)
Rigidity	4.30 (0.91)	24 (77)	—
Sleepiness (sedation)	4.30 (0.91)	25 (81)	—
Constipation	4.26 (0.90)	25 (81)	—
Itching	4.19 (0.83)	23 (74)	22 (81)
Urinating difficulties: urinary incontinence[b,f]	4.15 (0.86)	24 (77)	25 (93)
Dry mouth	4.07 (0.96)	24 (77)	—
Sexual function disorders[b]	3.93 (1.07)	—	23 (85)
**Burden of risks for adverse drug effects (n=11)**			
Risk of bleeding	4.81 (0.56)	24 (77)	—
Sedation	4.78 (0.58)	23 (74)	27 (100)
Renal toxicity	4.78 (0.58)	22 (71)	27 (100)
Anticholinergic effect	4.70 (0.61)	23 (74)	27 (100)

Risk of seizures	4.67 (0.62)	24 (77)	—
QT-prolongation	4.67 (0.68)	24 (77)	—
Potassium balance	4.63 (0.63)	25 (81)	—
Orthostatism	4.63 (0.69)	23 (74)	26 (96)
Sodium balance	4.59 (0.64)	25 (81)	—
Serotonergic effect	4.56 (0.70)	24 (77)	—
Constipation	4.41 (0.84)	22 (71)	26 (96)
**Laboratory tests and other test results (n=12)**			
Blood pressure	4.85 (0.53)	29 (97)	—
Kidney function	4.81 (0.56)	28 (93)	—
Blood glucose	4.78 (0.58)	28 (93)	—
International normalized ratio (INR)	4.78 (0.58)	26 (87)	—
Electrolytes (eg, sodium and potassium)	4.70 (0.61)	28 (93)	—
Hemoglobin	4.70 (0.61)	28 (93)	—
Liver function (ALAT, ASAT, and GT)	4.59 (0.64)	27 (90)	—
Cholesterol	4.48 (0.80)	24 (80)	—
Orthostatism	4.44 (0.93)	27 (90)	—
Phosphate	4.33 (0.78)	22 (73)	24 (86)
Mini-mental state examination[b]	4.30 (0.95)	—	23 (82)
Calcium	4.22 (0.89)	24 (80)	—
**Medication adherence topics (n=12)**			
The medicine user does not follow the given medication instructions because they do not understand the instructions	4.50 (0.92)	23 (74)	25 (89)
The medicine user does not follow the given medication instructions because they do not remember to take the medicine	4.46 (0.92)	24 (77)	—
Difficulties with swallowing the medicine	4.46 (1.04)	25 (81)	—
The medicine user does not follow the given medication instructions knowingly or intentionally	4.43 (0.88)	23 (74)	26 (93)
Difficulties with drug dosing	4.39 (1.03)	23 (74)	24 (86)
The medicine user expresses concern about possible adverse effects	4.25 (1.11)	21 (68)	25 (89)
The medicine user does not feel that the medicine has any therapeutic effect	4.21 (1.10)	20 (65)	25 (89)
Financial problems in purchasing medicines[d]	4.07 (1.12)	17 (55)	22 (79)
The medicine user does not know the purpose of the medication	4.04 (1.04)	18 (58)	22 (79)
Difficulties with opening the medicine packages[d]	3.93 (1.30)	19 (61)	22 (79)
Difficulties splitting tablets in half	3.86 (1.30)	20 (65)	22 (79)
The medicine user does not follow the given medication instructions because the dosing schedule does not fit the daily routines	3.75 (1.21)	19 (61)	23 (82)
**Topics related to the use of intoxicants (n=9)**			
Knowledge or suspicion of drug use disorder	4.57 (0.74)	28 (90)	—
Alcohol Use Disorders Identification Test (AUDIT-C) scores for alcohol consumption[b]	4.43 (0.79)	—	24 (86)
Information on what other intoxicants the person is using	4.36 (0.73)	24 (77)	—

Information on smoking and snuff use: quantity per day	4.14 (0.97)	25 (81)	—
Alcohol use: alcohol doses per week	4.04 (1.07)	24 (77)	—
Information on how often other intoxicants are used	3.93 (0.98)	22 (71)	23 (82)
Information on the quantity of use of other intoxicants	3.89 (0.96)	22 (71)	23 (82)
Performed AUDIT-C	3.89 (1.13)	27 (87)	—
Alcohol use: distribution of alcohol consumption according to weekdays	3.29 (1.18)	17 (55)	24 (86)
**Topics for which consensus was not reached (n=3)**			
Entitlement to reimbursement of medicine expenses	—	20 (64)	18 (64)
Drug shortages	—	19 (61)	18 (64)
Number of emergency department visits related to the use of intoxicants, if reliable information is not available in any other way[b]	—	—	17 (61)

[a]Not applicable

[b]Topics (n=8) suggested by expert panelists and added to the second Delphi round.

[c]Topic added by the research group to the third Delphi round.

[d]On the basis of the research group's decision, "Yes, but modified" responses (n=3) were included in consensus reached responses.

[e]Due to technical issues, this topic was not prioritized in the third Delphi round.

[f]The topic was specified and divided into 2 topics and repeated in the second round even though consensus was reached in the first round.
